# Association between the gut microbiota, inflammatory factors, and colorectal cancer: evidence from Mendelian randomization analysis

**DOI:** 10.3389/fmicb.2024.1309111

**Published:** 2024-03-18

**Authors:** Mingwei Ma, Zicheng Zheng, Jie Li, Yixuan He, Weiming Kang, Xin Ye

**Affiliations:** Department of General Surgery, Peking Union Medical College Hospital, Chinese Academy of Medical Science and Peking Union Medical College, Beijing, China

**Keywords:** gut microbiota, inflammatory factors, colorectal cancer, Mendelian randomization, cancer prevention

## Abstract

**Background:**

Colorectal cancer (CRC) is one of the most common malignant tumors primarily affecting individuals over the age of 50 years. Recent studies have suggested that the dysbiosis of the gut microbiota, a community of microorganisms in the human gut, is closely associated with the occurrence and development of CRC. Additionally, inflammatory factors (IFs) have also been reported to play a significant role in the development of CRC. However, the causal relationships between the gut microbiota, IFs, and CRC remain unclear.

**Methods:**

In this study, we performed Mendelian randomization (MR) analysis using publicly available genome-wide association study (GWAS) data to explore the causal relationship between the gut microbiota, IFs, and CRC. The gut microbiota GWAS data were obtained from the MiBioGen study, while the IFs GWAS data were derived from the comprehensive analysis of three independent cohorts. Causal relationship analysis was conducted using appropriate instrumental variables (IVs) and statistical models.

**Results:**

MR analysis of the gut microbiota and CRC revealed a negative correlation between the *Lachnospiraceae* species in the gut and CRC risk, while a positive correlation was observed between *Porphyromonadaceae* species, *Lachnospiraceae* UCG010 genus, *Lachnospira* genus, and *Sellimonas* genus in the gut, and CRC risk. Additionally, we observed a causal relationship between IL-10 and CRC risk. These findings suggest that the dysbiosis of the gut microbiota might be associated with an increased risk of CRC and that specific bacterial groups may play a crucial role in the occurrence and development of CRC.

**Conclusion:**

Using MR analysis, this study revealed the causal relationships between the gut microbiota, IFs, and CRC. The negative correlation between the *Lachnospiraceae* species in the gut and CRC risk, as well as the causal relationship between IL-10 and CRC, provide important clues for the potential roles of gut microbiota regulation and inflammatory factor control in the prevention and treatment of CRC.

## 1 Introduction

Colorectal cancer (CRC), a malignant tumor originating in the cells of the colon, is a common cancer typically occurring in individuals aged 50 years and above (Benson et al., 2018; Fabregas et al., 2022). Although the symptoms of CRC vary from person to person, some of the most common symptoms include abdominal pain and discomfort, changes in bowel habits (such as constipation, diarrhea, or increased frequency of bowel movements), presence of blood (either bright or dark red) in the stool, and intestinal obstruction (caused in the advanced stages of CRC when the tumor blocks the intestine, leading to severe abdominal pain, vomiting, and constipation) (Otani et al., 2019; Vogel et al., 2022). The risk factors for CRC include age (more common in individuals aged ≥50 years), genetic factors (individuals with a family history of CRC), gastrointestinal diseases (such as inflammatory bowel disease and familial adenomatous polyposis), high-fat, low-fiber diets, obesity, and diabetes (Giovannucci, 2002; Roslan et al., 2019).

The gut microbiota, which includes bacteria, archaea, viruses, fungi, protozoa, and parasites, plays a crucial role in the development of CRC. Recent research has shown a strong association between gut dysbiosis (imbalanced gut microbiota) and CRC (Garrett, 2019; Bai et al., 2022). Dysbiosis can lead to a reduction in the number of beneficial bacteria and an increase in the count of harmful bacteria, thereby disrupting the balance in the gut microbiota. This imbalance in the gut microbiota can lead to the production of harmful metabolites, such as carcinogens and inflammatory mediators, further promoting the development of CRC (Yang et al., 2022). Dysbiosis can also damage the intestinal mucosal barrier, allowing harmful substances and bacterial toxins to enter the intestinal tissue, thereby triggering an inflammatory response that promotes tumor formation and provides a favorable environment for tumor growth and metastasis (Wong and Yu, 2023).

Dysbiosis is also associated with changes in the tumor microenvironment of CRC (Zheng et al., 2020). Previous studies have suggested an association between specific groups of bacteria in the gut microbiome and CRC occurrence. For example, enrichment of the human gut with bacteria from the *Alistipes* genus has been associated with the development of CRC. These bacteria produce harmful metabolites (Louis et al., 2014), such as nitrosamines (Zhao et al., 2022), which promote the development of CRC (Parker et al., 2020). Therefore, the regulation of the gut microbiota serves as one of the potential strategies for the prevention and treatment of colon cancer (O'Keefe, 2016). Regulation of the composition and function of the gut microbiota can enhance the microbial balance in the gut by reducing the number of harmful bacteria and increasing the number of beneficial bacteria, thereby reducing the risk of CRC (Eslami et al., 2019). Some studies have shown that dietary changes, the use of probiotics and prebiotics, etc., regulate the gut microbiota and aid in the prevention and treatment of CRC (Tomasello et al., 2016; Pushpanathan et al., 2019).

Research has shown that the dysbiosis of the gut microbiota and the resulting inflammatory response play an important role in the occurrence and development of CRC (Fiorentini et al., 2020). Dysbiosis regulates the expression of the host genes associated with inflammation in the gut (Fidelle et al., 2020). Previous studies have shown that the dysbiosis of the gut microbiota can lead to the overexpression of inflammation-related genes, further exacerbating inflammatory responses and promoting the occurrence and development of colon cancer (Fidelle et al., 2020; Hou et al., 2022). Therefore, strategies aimed at regulating the gut microbiota may have the potential to modulate inflammatory responses. Mendelian randomization (MR), a relatively new technique that uses single nucleotide polymorphisms (SNPs) with an associated risk factor as instrumental variables (IVs), is used to determine if a causal relationship exists between a risk factor and a specific disease (Bowden and Holmes, 2019). Since the genetic variations detected in the zygote remain unchanged throughout life, these can be used in MR studies to avoid potential confounding variables or other sources of bias (Birney, 2022). In this study, we aimed to explore the causal relationship between the gut microbiota, inflammatory factors (IFs), and CRC, through the MR analysis of the summary-level data from publicly available genome-wide association studies (GWAS).

## 2 Materials and methods

### 2.1 Genome-wide association study data

Gut microbiota GWAS data were obtained from the MiBioGen study^1^, which is the most extensive multi-racial study on the gut microbiota thus far. In this study, the fecal microbiota data (*n* = 340) and the 16S genotyping data from 16 cohorts (*n* = 24,000) were analyzed to identify the relationship between the gut microbiota and human health. The results showed significant variations in the human gut microbiota across regions, ethnicities, and age groups. The genetic predictors of 41 systemic inflammatory regulators were obtained from a comprehensive cytokine-related GWAS meta-analysis conducted on three independent cohorts. These cohorts included 8,293 Finnish participants from the Cardiovascular Risk in Young Finns Study (YFS) and the “FINRISK” studies (FINRISK1997 and FINRISK2002) (Wang et al., 2022). To normalize the distributions of the 41 cytokines, a two-step inverse transformation was applied.

In order to test the univariable associations between 10.7 million genetic polymorphisms and the concentrations of the 41 cytokines, an additive genetic model was employed. This model took into account adjustments for age, sex, body mass index (BMI), and the first 10 genetic principal components. Lastly, the outcome data were obtained from the Finngen database.

### 2.2 Selection of instrumental variables

Bacterial classification and analyses were performed at five major taxonomic levels (phylum, class, order, family, and genus). To ensure the accuracy and validity of the causal relationships between the gut microbiota and CRC risk, we added restrictions to the IV inclusion criterion as follows. First, only the SNPs with *p* < 1e-05 were included as IVs for exposure and outcome analysis in the MR studies. Second, the TwoSampleMR R package was used to assign *r*^2^ = 0.001 and kb = 10,000 to ensure the independence of the selected IVs and to minimize the linkage disequilibrium effect that violates random allele assignment.

### 2.3 Statistical analysis

Mendelian randomization (MR) is a method used to investigate causal relationships between a modifiable exposure and an outcome using genetic instruments. There are two key assumptions in MR: assumption 1 states that the genetic instruments are associated with the exposure of interest, and assumption 2 states that any association between the instruments and the outcome is mediated by the exposure (Smith and Ebrahim, 2003). To address these assumptions, five MR methods were used in the analysis. The ratio method involved obtaining individual SNP estimates by dividing the SNP’s effect on schizophrenia by its corresponding effect on the biomarker. Standard errors were estimated assuming no measurement error. These estimates were then used for weighted analyses using other methods. Inverse variance weighting (IVW) is a commonly used method in MR (Burgess et al., 2013, 2017). It calculates the inverse variance weighted mean of ratio estimates from multiple instruments. This method assumes that all SNPs are valid instruments or that any bias is balanced across the instruments. Both fixed and random effects IVW methods were used. Weighted generalized linear regression is similar to the IVW method but allows for accounting for the correlation between genetic instruments. It was used when utilizing a conservative set of genetic instruments. The weighted median method calculates the median of the weighted empirical distribution function of individual SNP ratio estimates. This method provides a consistent effect estimate if more than 50% of the information comes from valid SNPs. Mendelian randomization Egger regression is a method that performs a weighted linear regression of SNP schizophrenia against SNP biomarker effect estimates (Bowden et al., 2015). It assumes that horizontal pleiotropic effects and SNP exposure associations are uncorrelated. The intercept of the MR Egger regression can be interpreted as a test for overall unbalanced horizontal pleiotropy. Both fixed and random effects versions of this method were performed. By employing these five MR methods, the researchers aimed to minimize bias and obtain reliable estimates of the causal relationship between the modifiable exposure and the outcome of interest. Different causality analysis models were used in this study. Among them, the inverse-variance weighted (IVW) model and MR-Egger method were used for the analysis of samples with multiple SNPs, while the Wald ratio test was used for the analysis of samples with only one SNP.

For sensitivity analyses, heterogeneity was measured using the Cochran Q method. In case of obvious heterogeneity (*p* < 0.05), MR-Egger regression analysis was used to assess the potential pleiotropic inheritance of the SNPs used as IVs. In MR-Egger regression, the intercept term indicates directed horizontal pleiotropy at *p* < 0.05. All statistical analyses in this study were performed using the R package in the R language application (v4.2.1).

## 3 Results

### 3.1 Mendelian randomization analysis of the gut microbiota and colorectal cancer

**Figure 1:**
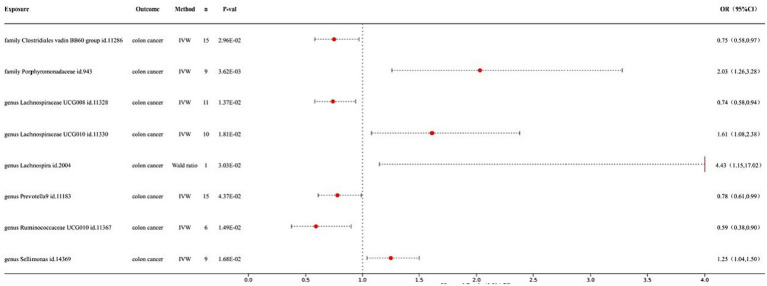
Forest map of MR results of gut microbiota and colon cancer.

Our preliminary study revealed that 8 out of the 211 gut bacteria may have a causal relationship with CRC (Figure 1). The IVW analysis results for these 8 bacteria were as follows: family *Clostridiales vadin* BB60 group id.11286 (*p* = 2.96E-02; odds ratio (Fabregas et al., 2022) 95% confidence interval (Benson et al., 2018) = 0.75 (0.58, 0.97)), family *Porphyromonadaceae* id.943 (*p* = 3.62E-03; OR 95% CI = 2.03 (1.26, 3.28)), genus *Lachnospiraceae* UCG008 id.11328 (*p* = 1.37E-02; OR 95% CI = 0.74 (0.58, 0.94)), genus *Lachnospiraceae* UCG010 id.11330 (*p* = 1.81E-02; OR 95% CI = 1.61 (1.08, 2.38)), genus *Lachnospira* id.2004 (*p* = 3.03E-02; OR 95% CI = 4.43 (1.15, 17.02)), genus *Prevotella* 9 id.11183 (*p* = 4.37E-02; OR 95% CI = 0.78 (0.61, 0.99)), genus *Ruminococcaceae* UCG010 id.11367 (*p* = 1.49E-02; OR 95% CI = 0.59 (0.38, 0.90)), and genus *Sellimonas* id.14369 (*p* = 1.68E-02; OR 95% CI = 1.25 (1.04, 1.50)). Among them, family *Porphyromonadaceae* id.943, genus *Lachnospiraceae* UCG010 id.11330, genus *Lachnospira* id.2004, and genus *Sellimonas* id.14369 showed a positive correlation with CRC risk, while the other bacterial classes showed a negative correlation, indicating their protective effects. Detailed information on the MR analysis of the gut microbiota and CRC can be found in the Supplementary material S1.

### 3.2 Mendelian randomization analysis of inflammatory factors and colorectal cancer

**Figure 2:**
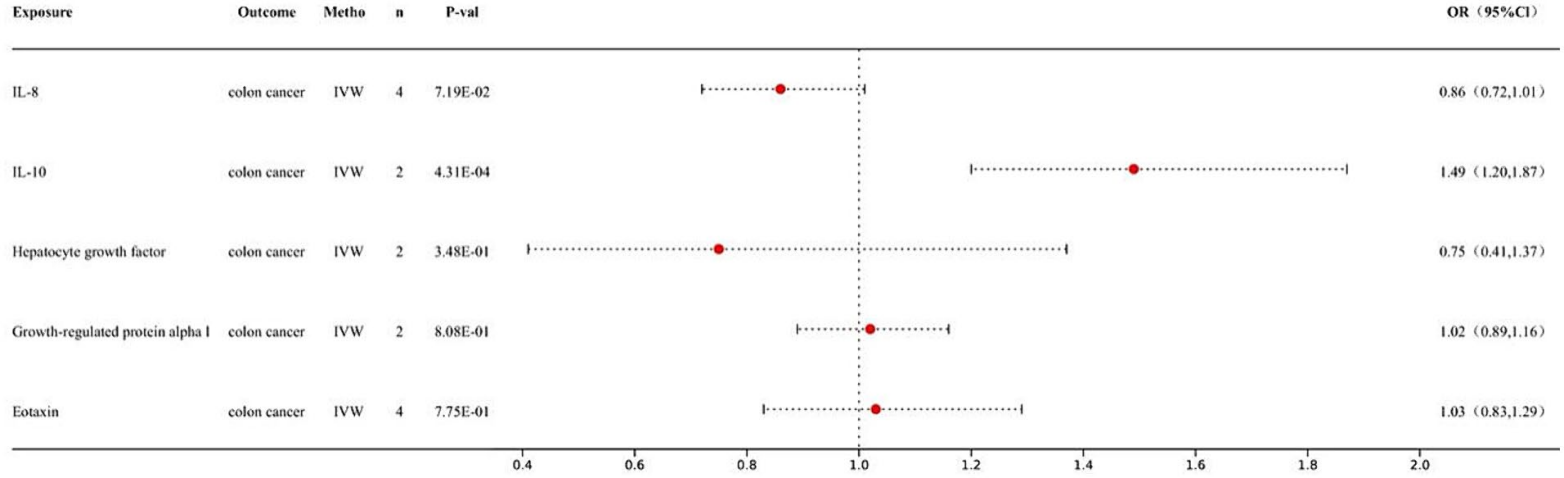
Forest map of MR results of inflammatory factors and colon cancer.

This study revealed a causal relationship between one of the 41 inflammatory factors and CRC (Figure 2). The results obtained from the IVW analysis of interleukin-10 and CRC were as follows: (*p* = 4.31E-04; OR 95% CI = 1.49 (1.20, 1.87)). Detailed information on the MR analysis of the gut microbiota and CRC and the inflammatory factors and CRC can be found in the Supplementary material S1.

### 3.3 Mendelian randomization analysis of the gut microbiota and inflammatory factors

**Figure 3:**
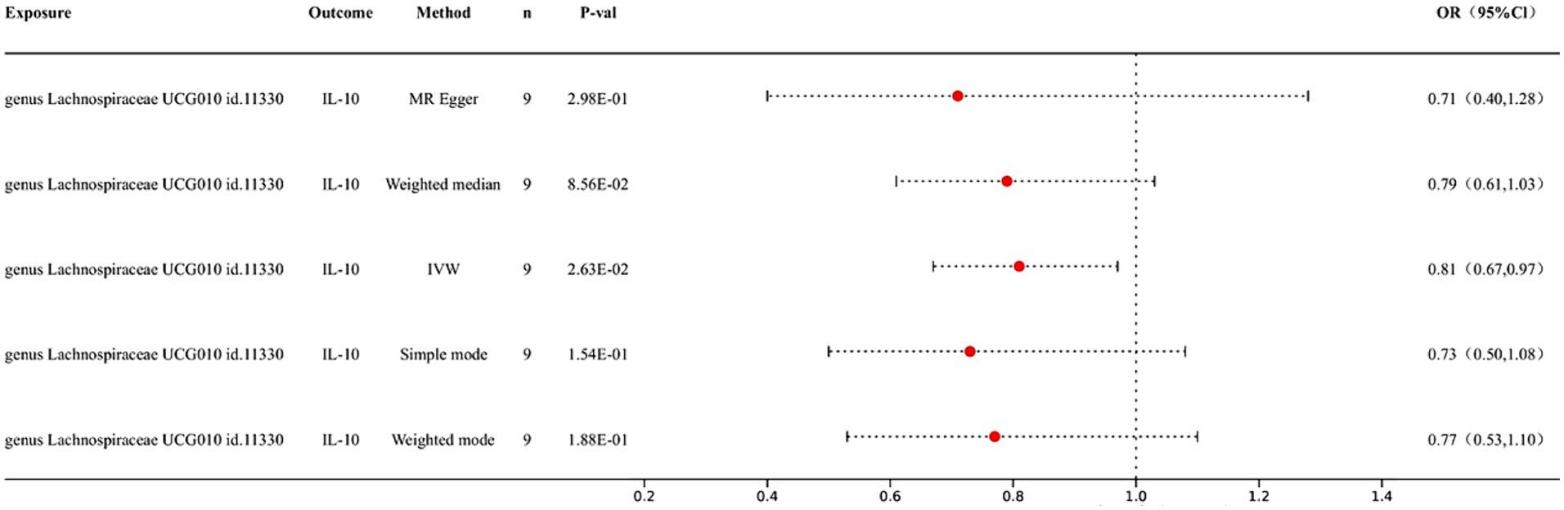
Forest map of MR results of gut microbiota and inflammatory factors.

We conducted an MR analysis of the gut microbiota and inflammatory factors to further elucidate the role of inflammatory factors in the association between gut microbiota and CRC. IVW analysis results showed a causal relationship between genus *Lachnospiraceae* UCG010 id.11330 and IL-10 (*p* = 2.63E-02; OR 95% CI = 0.81 (0.67, 0.97)); no significant association was observed between any of the other bacterial taxa and inflammatory factors (Figure 3).

**Figure 4:**
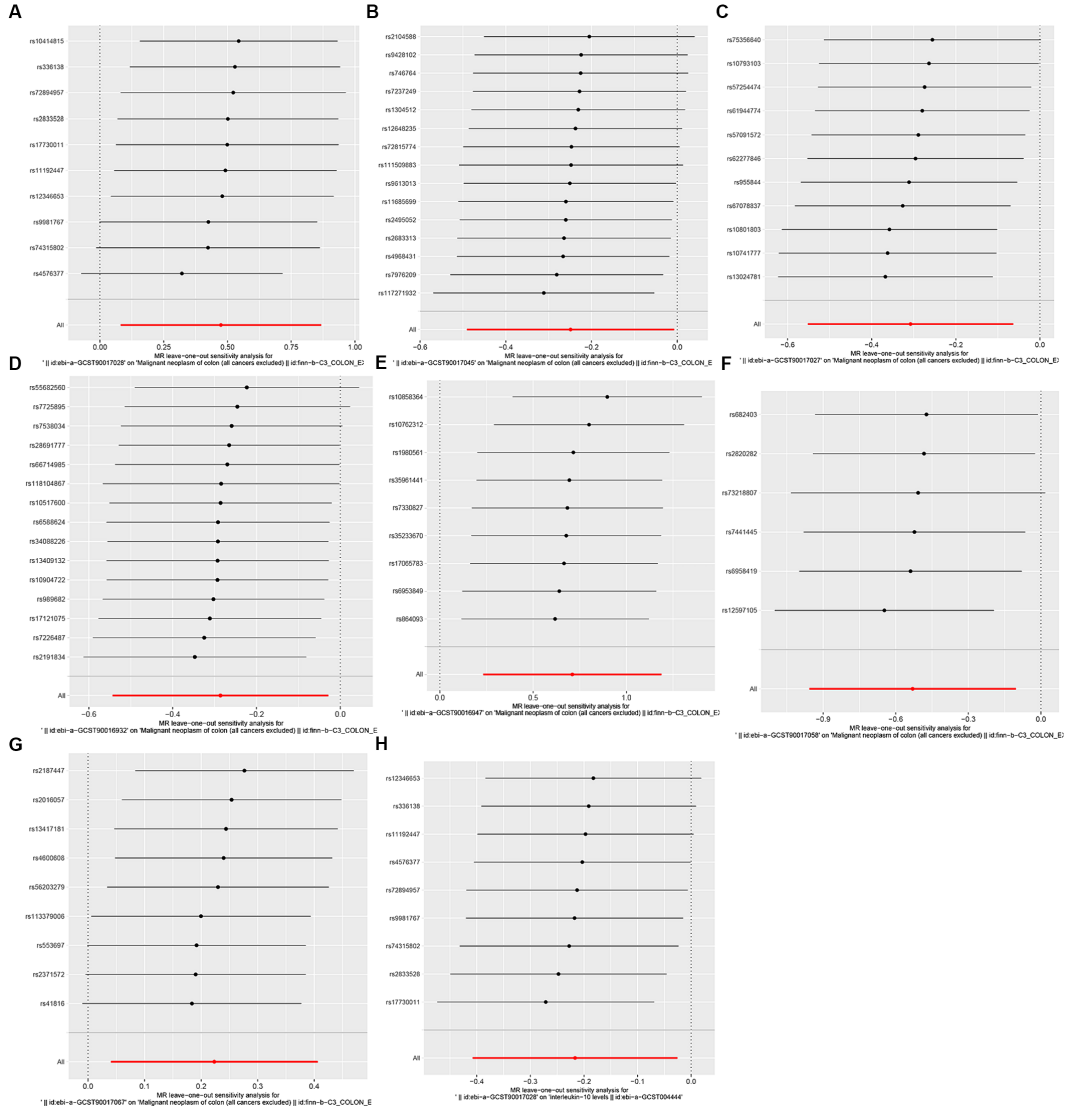
leave-one-out plot. **(A)** Leave-one-out plot of genus Lachnospiraceae UCG010 id.11330 and CRC, **(B)** Leave-one-out plot of genus Prevotella9 id.11183 and CRC; **(C)** Leave-one-out plot ofgenus Lachnospiraceae UCG008 id.11328 and CRC; **(D)** Leave-one-out plot of genusfamily Clostridiales vadin BB60 group id.11286 and CRC; **(E)** Leave-one-out plot of family Porphyromonadaceae id.943 and CRC; **(F)** Leave-one-out plot of genus Ruminococcaceae UCG010 id.11367 and CRC; **(G)** Leave-one-out plot of genus Sellimonas id.14369 and CRC; **(H)** Leave-one-out plot of genus Lachnospiraceae UCG010 id.11330 and IL-10.

In sensitivity analysis, we conducted heterogeneity and pleiotropy analyses for the immune cells included in our study and their respective diseases. Our results all yielded *p*-values greater than 0.05, indicating the absence of heterogeneity and pleiotropy SNPs. Additionally, we performed leave-one-out analysis, which also demonstrated the stability of our results. The leave-one-out plot is Figure 4, while the heterogeneity results are presented in Table 1 and the pleiotropy analysis results in Table 2.

**Table 1:** The heterogeneity test of gut microbiota, inflammatory factors, and colorectal cancer in this study.

id.exposure	Outcome	Method	Q	Q_df	Q_pval
family Clostridiales vadin BB60 group id.11286	CRC	MR Egger	8.31	13	0.82
family Clostridiales vadin BB60 group id.11286	CRC	IVW	10.41	14	0.73
family Porphyromonadaceae id.943	CRC	MR Egger	6.85	7	0.44
family Porphyromonadaceae id.943	CRC	IVW	7.17	8	0.52
genus Lachnospiraceae UCG008 id.11328	CRC	MR Egger	7.36	9	0.60
genus Lachnospiraceae UCG008 id.11328	CRC	IVW	9.20	10	0.51
genus Lachnospiraceae UCG010 id.11330	CRC	MR Egger	7.86	8	0.45
genus Lachnospiraceae UCG010 id.11330	CRC	IVW	9.85	9	0.36
genus Prevotella9 id.11183	CRC	MR Egger	9.08	13	0.77
genus Prevotella9 id.11183	CRC	IVW	9.49	14	0.80
genus Ruminococcaceae UCG010 id.11367	CRC	MR Egger	2.65	4	0.62
genus Ruminococcaceae UCG010 id.11367	CRC	IVW	2.65	5	0.75
genus Sellimonas id.14369	CRC	MR Egger	5.86	7	0.56
genus Sellimonas id.14369	CRC	IVW	7.26	8	0.51
genus Lachnospiraceae UCG010 id.11330	Interleukin-10	MR Egger	5.00	7	0.66
genus Lachnospiraceae UCG010 id.11330	Interleukin-10	IVW	5.18	8	0.74
Interleukin-10	CRC	IVW	0.00	1	0.97

**Table 2:** The pleiotropy test of gut microbiota, inflammatory factors, and colorectal cancer in this study could not be conducted for some immune cells due to insufficient SNPs being included.

id.exposure	id.outcome	egger_intercept	se	pval
family Clostridiales vadin BB60 group id.11286	CRC	0.05	0.03	0.17
family Porphyromonadaceae id.943	CRC	−0.04	0.06	0.59
genus Lachnospiraceae UCG008 id.11328	CRC	0.09	0.07	0.21
genus Lachnospiraceae UCG010 id.11330	CRC	0.06	0.04	0.20
genus Prevotella9 id.11183	CRC	−0.02	0.04	0.53
genus Ruminococcaceae UCG010 id.11367	CRC	0.00	0.04	0.97
genus Sellimonas id.14369	CRC	0.09	0.08	0.28
genus Lachnospiraceae UCG010 id.11330	Interleukin-10	0.01	0.02	0.69
Interleukin-10	CRC	NA	NA	NA

## 4 Discussion

In this study, we conducted a dual sample MR analysis to investigate the causal relationship between gut microbiota, inflammatory factors, and CRC. We found a potential causal relationship between the Lachnospiraceae UCG010 id.11330 bacterial genus and IL-10, CRC. The results showed that Lachnospiraceae UCG010 id.11330 increased the incidence of CRC, and IL-10 also increased the incidence of CRC. However, further investigation indicated a negative correlation between Lachnospiraceae UCG010 id.11330 and IL-10. Based on these findings, it is hypothesized that the increase in CRC caused by Lachnospiraceae UCG010 id.11330 is not mediated by IL-10. These two processes may be unrelated.

The association between the gut microbiota and CRC has been studied extensively and is supported by a substantial body of evidence. In this context, certain pathogenic bacteria can indirectly induce DNA damage in the host cells or interfere with important cell signaling pathways related to cell proliferation, apoptosis, and inflammation by producing enzymatically active protein toxins, thereby exerting a pro-tumorigenic effect (Chen and Li, 2020; Mirzaei et al., 2021). Bacteria are an important component of the gut microbiota, and several bacterial taxa harbor strains that produce protein toxins with potential pro-carcinogenic properties. Data on the consequences of long-term exposure to these gut bacteria and their toxins is gradually emerging, although research in this field is still relatively limited (Illescas et al., 2021). Previous studies have demonstrated that Lachnospiraceae UCG010 id.11330 is a potential biomarker closely related to oxidative stress and metabolic genes (Qin et al., 2022). Oxidative stress plays an important role in the initiation and promotion stage of colon cancer, which may be the reason for the increased risk of CRC caused by Lachnospiraceae UCG010 id.11330 (Miyamoto et al., 2019).

Inflammation is a significant factor in the development of CRC. Chronic inflammation can lead to abnormal cell proliferation and mutations, increasing the risk of developing cancer. Inflammation can also alter the intestinal microenvironment, promoting tumor growth and metastases (Shawki et al., 2018; Dong et al., 2019). Conditions such as ulcerative colitis (UC) and Crohn’s disease (CD) can cause chronic inflammation in the intestine, thereby increasing the risk of CRC. Patients with UC and CD have a higher incidence of CRC and require regular monitoring and screening. There is a complex interaction between inflammation and genetic factors (Goc et al., 2021). Inflammation can alter gene expression, leading to abnormal cell proliferation and mutations, and certain genetic mutations can increase the risk of developing CRC. The interaction between genetic factors and inflammation plays a crucial role in the development of CRC (Goc et al., 2021). There is a close relationship between inflammation and the immune system. Inflammation can activate the immune system, enhancing its ability to eliminate tumor cells. The expression and function of IL-10, an immune regulatory factor (Zegarra Ruiz et al., 2022) that has a significant impact on CRC development and treatment, have been studied extensively in CRC (Lian et al., 2019). Studies have shown that elevated levels of IL-10 in CRC tissues are closely associated with tumor staging, lymph node metastasis, and poor prognosis. Additionally, increased IL-10 expression is also associated with increased invasiveness and metastatic potential of the tumors (Lian et al., 2019). In CRC, IL-10 primarily affects tumor development by regulating immune and inflammatory responses. It inhibits the activation and functioning of the immune cells, thereby reducing tumor cell clearance by cytotoxic T cells and natural killer cells (Sethi et al., 2018). Furthermore, it suppresses inflammatory responses and cell apoptosis, thereby promoting tumor cell proliferation and survival. The application of IL-10 in CRC treatment is gaining great interest. Some studies have found that the inhibition of IL-10 expression or function enhances the killing effect exerted by the immune cells on the tumors, thereby improving treatment outcomes. Additionally, inhibiting IL-10 expression or function can also reduce tumor invasiveness and metastasis, thereby improving patient prognosis (Cai and Zhang, 2016; Rossowska et al., 2018; Huang et al., 2020).

The relationship between the gut microbiota and digestive tract cancer has been a topic of considerable interest. Increasing evidence suggests that the microbiota may play a significant role in the pathogenesis of digestive tract cancer, including influencing host immune responses, metabolite production, chronic inflammation, and intestinal mucosal barrier function (Zou et al., 2018; Fan et al., 2021; Lee et al., 2023). Factors such as inflammation and bacterial infection may cause a shift from the symbiotic state of the gut microbiota to a pro-carcinogenic configuration (Weinberg and Marshall, 2019). However, our study found a negative correlation between Lachnospiraceae UCG010 id.11330 and IL-10, suggesting that Lachnospiraceae UCG010 id.11330 does not mediate colon cancer through IL-10. Recent literature has reported that the abundance of LachnospiraceaeUCG-009 is negatively associated with inflammatory factors such as interleukin-12P40, interferon, and DR5 with specific bacterial genera (Xu et al., 2022). In addition, recent literature has reported that Lachnospiraceae UCG-006 may modulate the immune system and gut microbiota through its anti-allergic and anti-inflammatory effects, which also supports the possible anti-inflammatory effects of Lachnospiraceae (Li et al., 2022).

In recent decades, researchers have actively explored the potential connection between the gut microbiota and digestive tract cancer, seeking to understand the role of the microbiota in the occurrence, development, and treatment of cancer. MR is a method used to assess the effects of therapeutic interventions and is commonly employed in clinical trials. The relationship between the gut microbiota and digestive may be utilized to evaluate the impact of specific microbial communities or microbial combinations on the development and treatment of cancer. Numerous similar studies have demonstrated the significant role of MR in research on the gut microbiota and digestive tract cancer (Ni et al., 2022; Li et al., 2023; Long et al., 2023; Xie et al., 2023).

Conclusively, this study has several advantages over other similar studies: The use of Mendelian randomization analysis in this study effectively controlled for confounding factors, while leveraging a large-scale GWAS dataset enhanced the statistical power and generalizability of the findings. The exploration of the relationship between gut microbiota, inflammatory factors, and colorectal cancer not only sheds light on potential prevention and treatment strategies but also contributes to a deeper understanding of the underlying mechanisms. Furthermore, the identification of specific bacterial groups associated with colorectal cancer risk provides promising targets for future interventions and therapeutic approaches aimed at modulating the gut microbiota to mitigate CRC risk. However, it also has some limitations. Firstly, the results of this study can be applied only to specific populations and samples because the participants were predominantly of European descent. Additionally, potential variations in population characteristics and data collection methods exist. Despite efforts to gather data, the lack of comprehensive data hinders further statistical analysis to adjust for potential confounding factors, which is also a common challenge in Mendelian randomization studies. Secondly, gut microbes are diverse and complex, and their potential confounding factors may have some influence on causality. In the future, we will further design prospective controlled experiments to investigate the mechanism of action between gut microbiota and CRC.

## 5 Conclusion

There is a causal relationship between the gut microbiota, IL-10 and CRC. Regulation of the gut microbiota and anti-inflammatory ability may serve as a potential strategy for the prevention and treatment of CRC.

## Data availability statement

The original contributions presented in the study are included in the article/Supplementary material, further inquiries can be directed to the corresponding authors.

## Author contributions

MM: Conceptualization, Methodology, Writing – original draft. ZZ: Conceptualization, Methodology, Writing – review & editing. JL: Formal analysis, Methodology, Resources, Writing – review & editing. YH: Data curation, Methodology, Writing – original draft. WK: Funding acquisition, Writing – review & editing. XY: Funding acquisition, Investigation, Writing – review & editing.

## Footnotes

^1^https://mibiogen.rug.nl/
